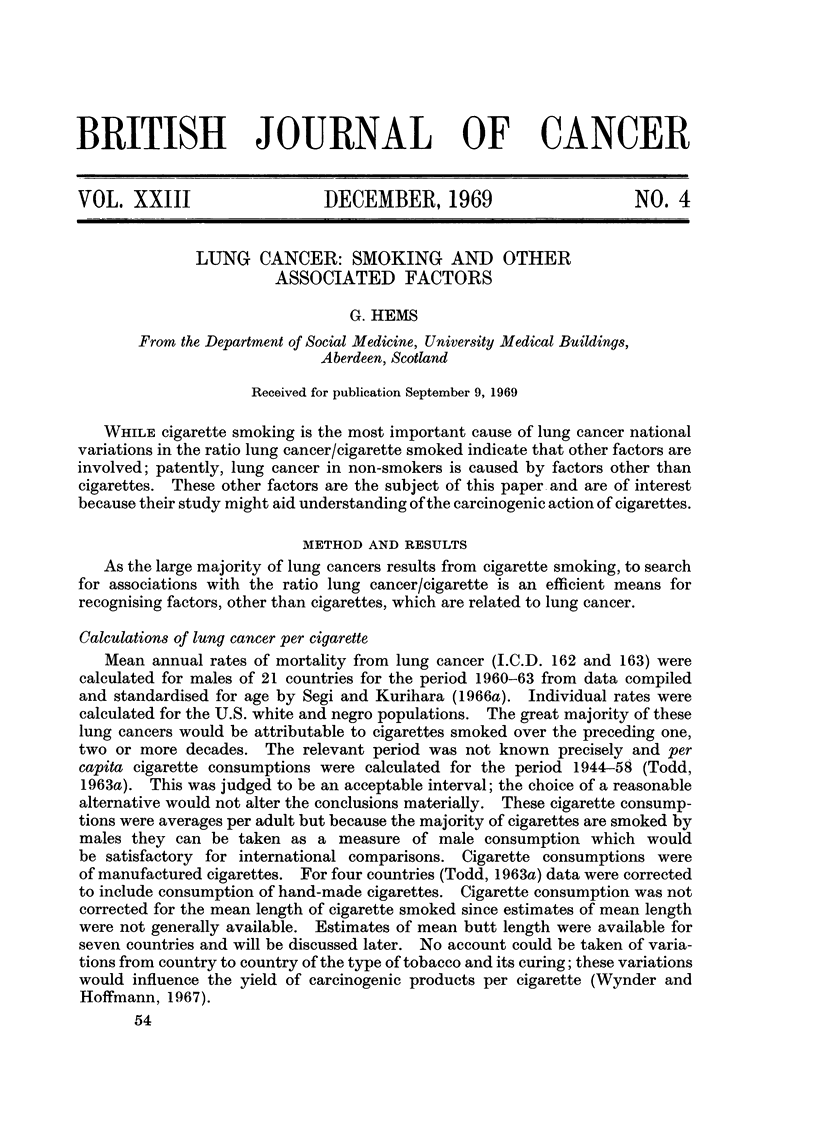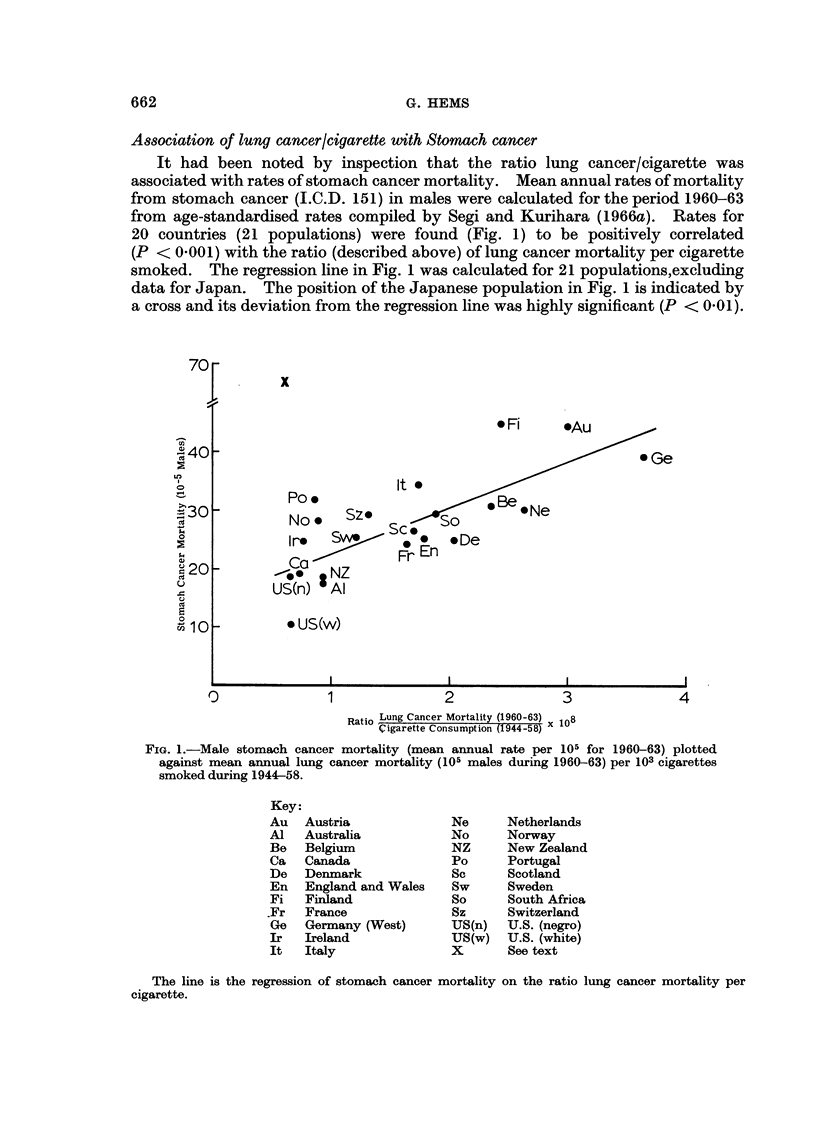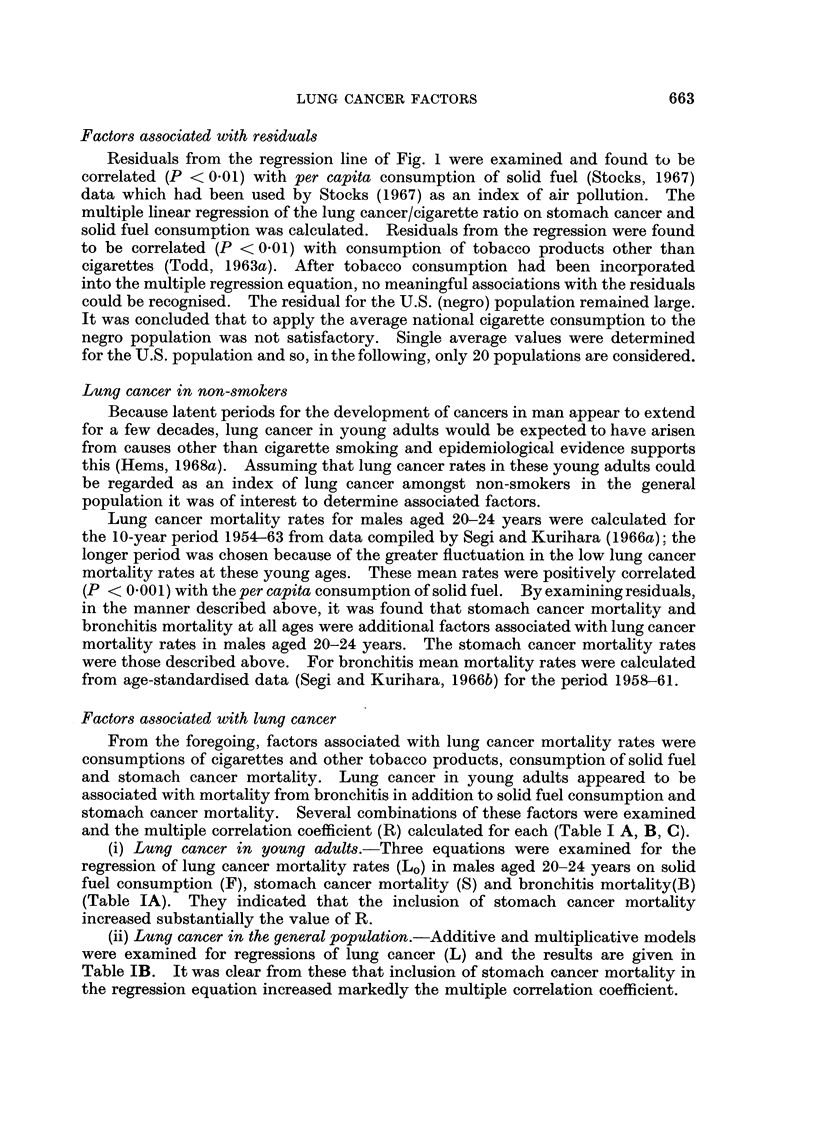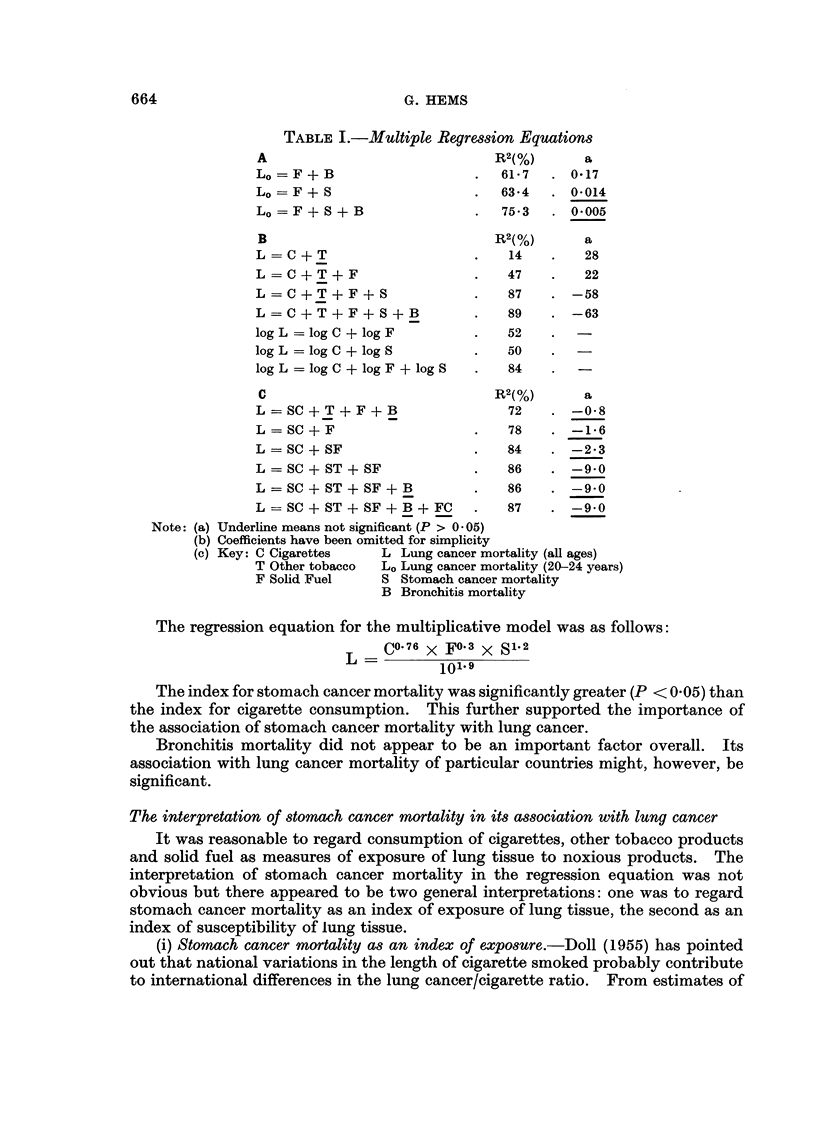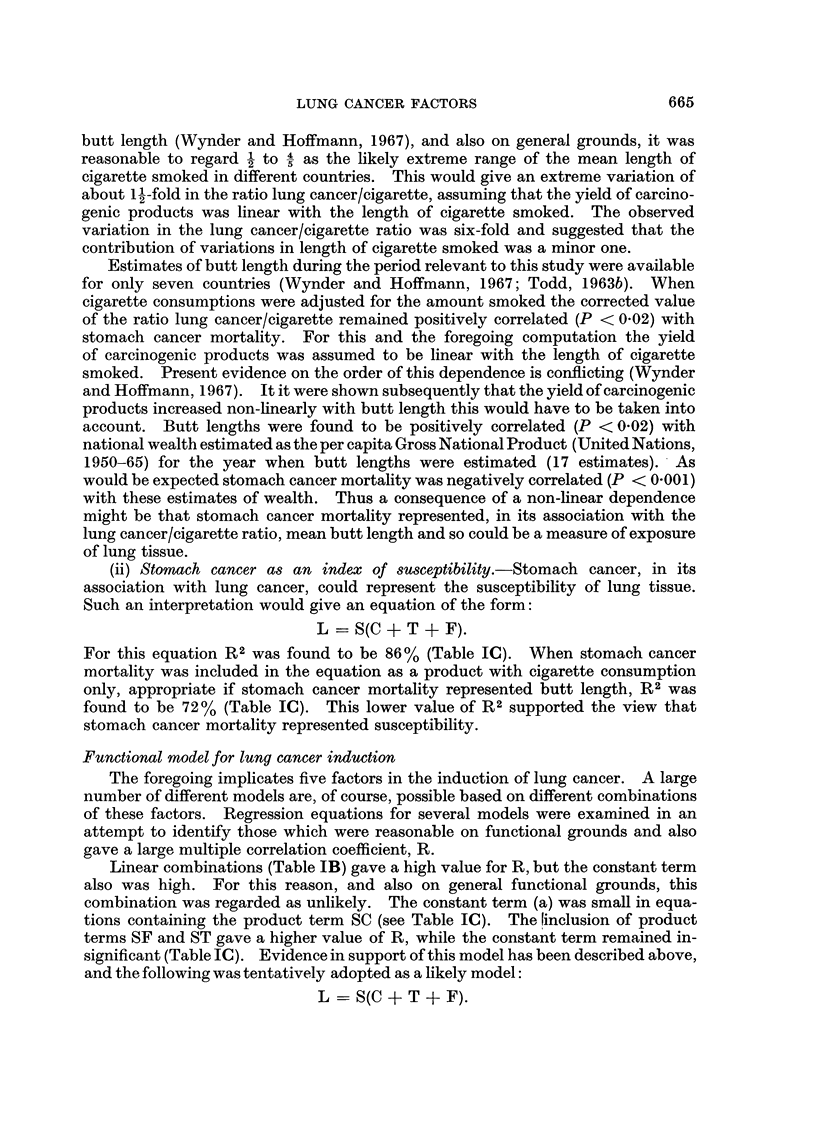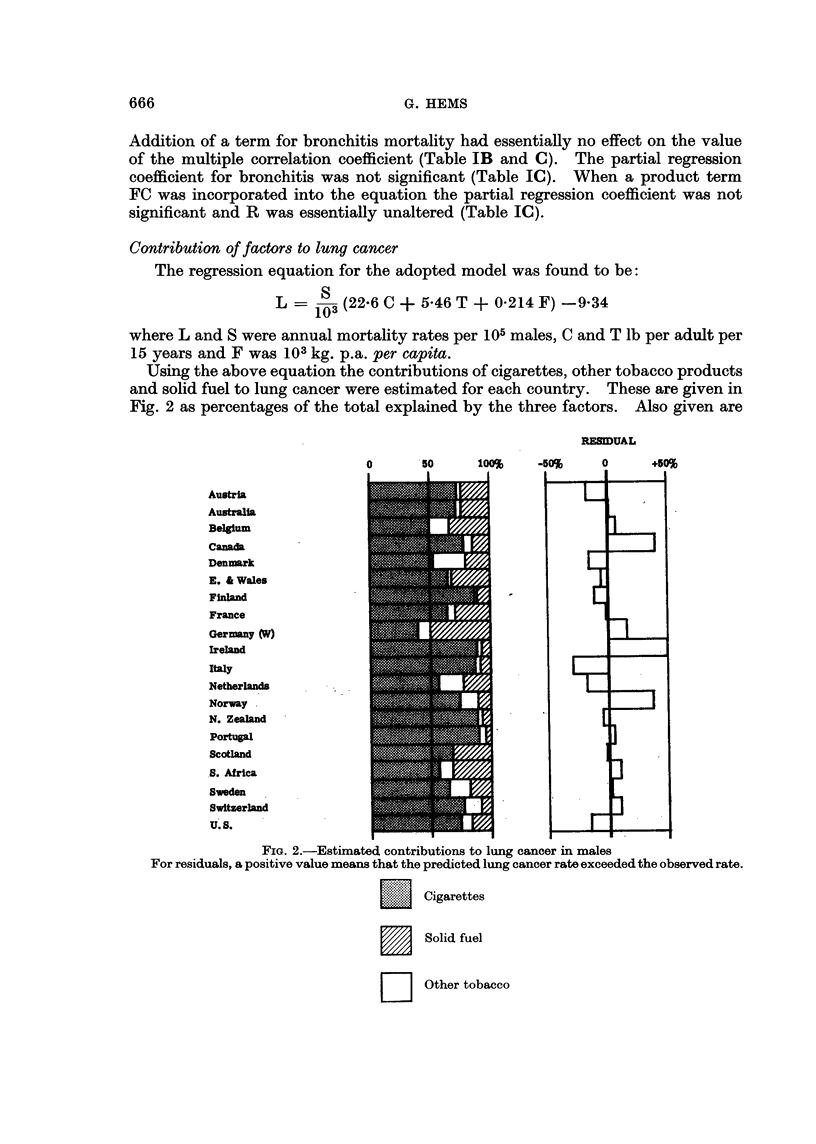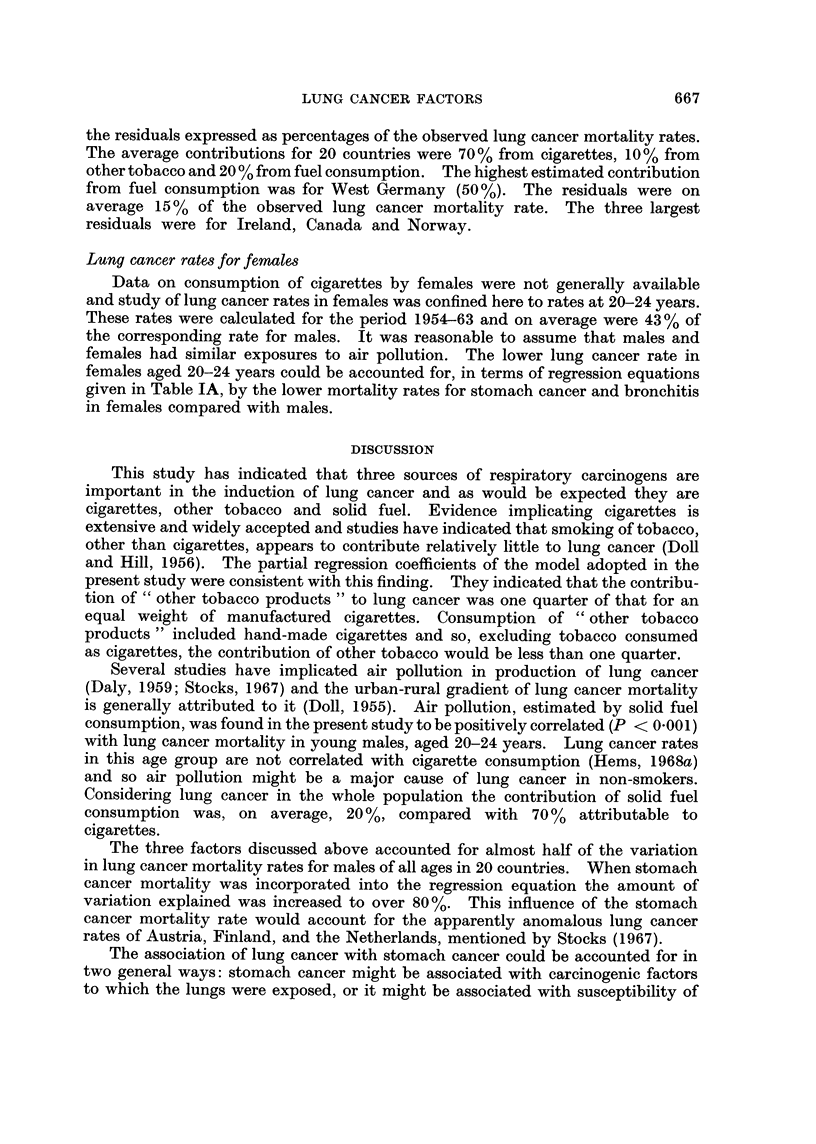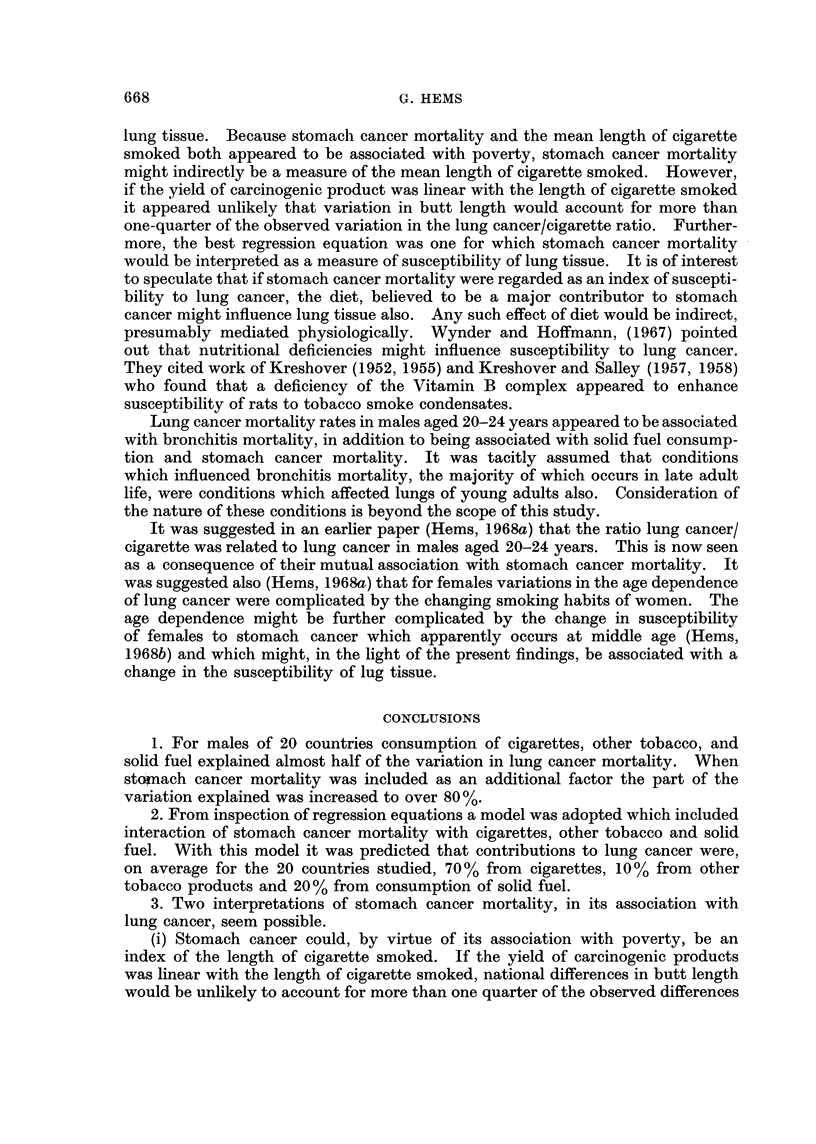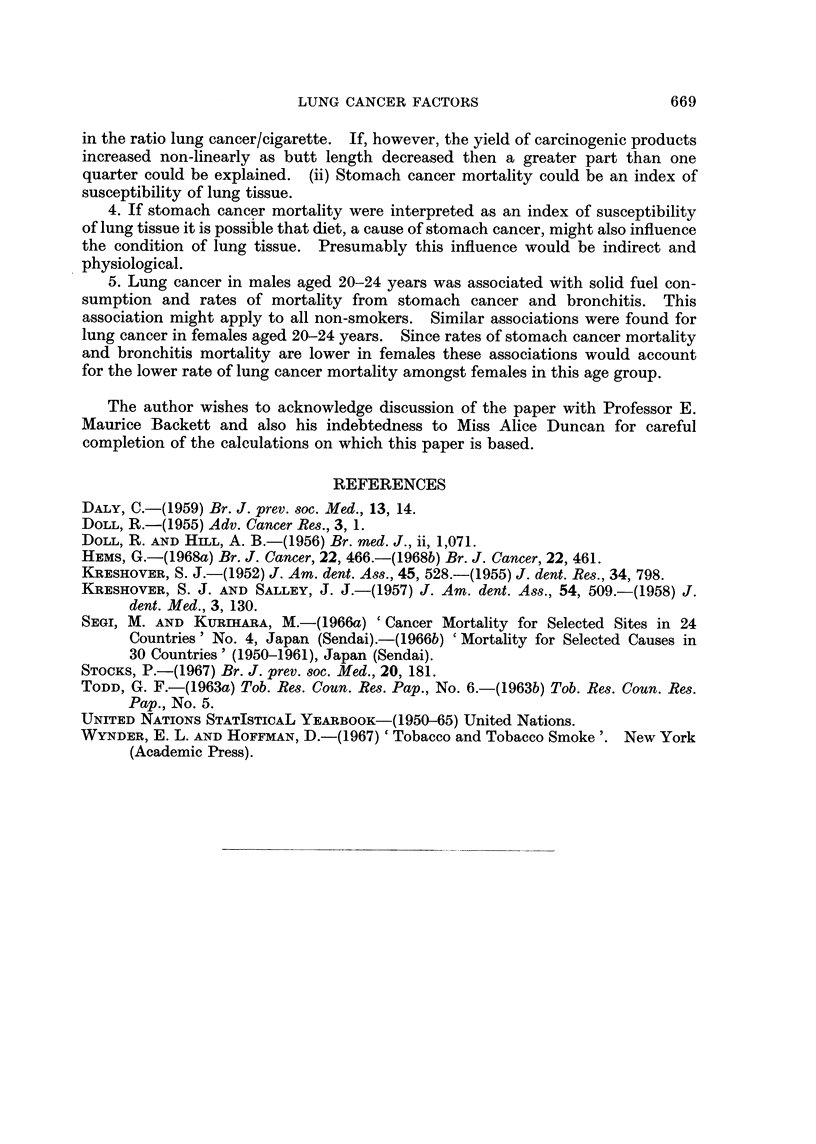# Lung cancer: smoking and other associated factors.

**DOI:** 10.1038/bjc.1969.82

**Published:** 1969-12

**Authors:** G. Hems


BRITISH JOURNAL OF CANCER

VOL. XXIII DECEMBER, 1969 NO. 4

### LUNG CANCER: SMOKING AND OTHER ASSOCIATED FACTORS

G. HEMS

*From the Department of Social Medicine, University Medical Buildings, Aberdeen, Scotland*

Received for publication September 9, 1969

WHILE cigarette smoking is the most important cause of lung cancer national variations in the ratio lung cancer/cigarette smoked indicate that other factors are involved; patently, lung cancer in non-smokers is caused by factors other than cigarettes. These other factors are the subject of this paper and are of interest because their study might aid understanding of the carcinogenic action of cigarettes.

### METHOD AND RESULTS

As the large majority of lung cancers results from cigarette smoking, to search for associations with the ratio lung cancer/cigarette is an efficient means for recognising factors, other than cigarettes, which are related to lung cancer.

### *Calculations of lung cancer per cigarette*

Mean annual rates of mortality from lung cancer (I.C.D. 162 and 163) were calculated for males of 21 countries for the period 1960–63 from data compiled and standardised for age by Segi and Kurihara (1966*a*). Individual rates were calculated for the U.S. white and negro populations. The great majority of these lung cancers would be attributable to cigarettes smoked over the preceding one, two or more decades. The relevant period was not known precisely and *per capita* cigarette consumptions were calculated for the period 1944–58 (Todd, 1963*a*). This was judged to be an acceptable interval; the choice of a reasonable alternative would not alter the conclusions materially. These cigarette consumptions were averages per adult but because the majority of cigarettes are smoked by males they can be taken as a measure of male consumption which would be satisfactory for international comparisons. Cigarette consumptions were of manufactured cigarettes. For four countries (Todd, 1963*a*) data were corrected to include consumption of hand-made cigarettes. Cigarette consumption was not corrected for the mean length of cigarette smoked since estimates of mean length were not generally available. Estimates of mean butt length were available for seven countries and will be discussed later. No account could be taken of variations from country to country of the type of tobacco and its curing; these variations would influence the yield of carcinogenic products per cigarette (Wynder and Hoffmann, 1967).

*Association of lung cancer/cigarette with Stomach cancer*

It had been noted by inspection that the ratio lung cancer/cigarette was associated with rates of stomach cancer mortality. Mean annual rates of mortality from stomach cancer (I.C.D. 151) in males were calculated for the period 1960–63 from age-standardised rates compiled by Segi and Kurihara (1966*a*). Rates for 20 countries (21 populations) were found (Fig. 1) to be positively correlated ($P < 0{\cdot}001$) with the ratio (described above) of lung cancer mortality per cigarette smoked. The regression line in Fig. 1 was calculated for 21 populations, excluding data for Japan. The position of the Japanese population in Fig. 1 is indicated by a cross and its deviation from the regression line was highly significant ($P < 0{\cdot}01$).

FIG. 1.—Male stomach cancer mortality (mean annual rate per $10^5$ for 1960–63) plotted against mean annual lung cancer mortality ($10^5$ males during 1960–63) per $10^3$ cigarettes smoked during 1944–58.

Key:

| | | | |
|---|---|---|---|
| Au | Austria | Ne | Netherlands |
| Al | Australia | No | Norway |
| Be | Belgium | NZ | New Zealand |
| Ca | Canada | Po | Portugal |
| De | Denmark | Sc | Scotland |
| En | England and Wales | Sw | Sweden |
| Fi | Finland | So | South Africa |
| .Fr | France | Sz | Switzerland |
| Ge | Germany (West) | US(n) | U.S. (negro) |
| Ir | Ireland | US(w) | U.S. (white) |
| It | Italy | X | See text |

The line is the regression of stomach cancer mortality on the ratio lung cancer mortality per cigarette.

*Factors associated with residuals*

Residuals from the regression line of Fig. 1 were examined and found to be correlated ($P < 0 \cdot 01$) with *per capita* consumption of solid fuel (Stocks, 1967) data which had been used by Stocks (1967) as an index of air pollution. The multiple linear regression of the lung cancer/cigarette ratio on stomach cancer and solid fuel consumption was calculated. Residuals from the regression were found to be correlated ($P < 0 \cdot 01$) with consumption of tobacco products other than cigarettes (Todd, 1963*a*). After tobacco consumption had been incorporated into the multiple regression equation, no meaningful associations with the residuals could be recognised. The residual for the U.S. (negro) population remained large. It was concluded that to apply the average national cigarette consumption to the negro population was not satisfactory. Single average values were determined for the U.S. population and so, in the following, only 20 populations are considered.

*Lung cancer in non-smokers*

Because latent periods for the development of cancers in man appear to extend for a few decades, lung cancer in young adults would be expected to have arisen from causes other than cigarette smoking and epidemiological evidence supports this (Hems, 1968*a*). Assuming that lung cancer rates in these young adults could be regarded as an index of lung cancer amongst non-smokers in the general population it was of interest to determine associated factors.

Lung cancer mortality rates for males aged 20–24 years were calculated for the 10-year period 1954–63 from data compiled by Segi and Kurihara (1966*a*); the longer period was chosen because of the greater fluctuation in the low lung cancer mortality rates at these young ages. These mean rates were positively correlated ($P < 0 \cdot 001$) with the *per capita* consumption of solid fuel. By examining residuals, in the manner described above, it was found that stomach cancer mortality and bronchitis mortality at all ages were additional factors associated with lung cancer mortality rates in males aged 20–24 years. The stomach cancer mortality rates were those described above. For bronchitis mean mortality rates were calculated from age-standardised data (Segi and Kurihara, 1966*b*) for the period 1958–61.

*Factors associated with lung cancer*

From the foregoing, factors associated with lung cancer mortality rates were consumptions of cigarettes and other tobacco products, consumption of solid fuel and stomach cancer mortality. Lung cancer in young adults appeared to be associated with mortality from bronchitis in addition to solid fuel consumption and stomach cancer mortality. Several combinations of these factors were examined and the multiple correlation coefficient (R) calculated for each (Table I **A**, **B**, **C**).

(i) *Lung cancer in young adults.*—Three equations were examined for the regression of lung cancer mortality rates ($L_0$) in males aged 20–24 years on solid fuel consumption (F), stomach cancer mortality (S) and bronchitis mortality(B) (Table I**A**). They indicated that the inclusion of stomach cancer mortality increased substantially the value of R.

(ii) *Lung cancer in the general population.*—Additive and multiplicative models were examined for regressions of lung cancer (L) and the results are given in Table I**B**. It was clear from these that inclusion of stomach cancer mortality in the regression equation increased markedly the multiple correlation coefficient.

TABLE I.—*Multiple Regression Equations*

| A | $R^2$(%) | a |
|---|---|---|
| $L_0 = F + B$ | 61·7 | 0·17 |
| $L_0 = F + S$ | 63·4 | $\underline{0{\cdot}014}$ |
| $L_0 = F + S + B$ | 75·3 | $\underline{0{\cdot}005}$ |
| **B** | $R^2$(%) | a |
| $L = C + \underline{T}$ | 14 | 28 |
| $L = C + \underline{T} + F$ | 47 | 22 |
| $L = C + \underline{T} + F + S$ | 87 | −58 |
| $L = C + T + F + S + \underline{B}$ | 89 | −63 |
| $\log L = \log C + \log F$ | 52 | — |
| $\log L = \log C + \log S$ | 50 | — |
| $\log L = \log C + \log F + \log S$ | 84 | — |
| **C** | $R^2$(%) | a |
| $L = SC + \underline{T} + F + \underline{B}$ | 72 | $\underline{-0{\cdot}8}$ |
| $L = SC + F$ | 78 | $\underline{-1{\cdot}6}$ |
| $L = SC + SF$ | 84 | $\underline{-2{\cdot}3}$ |
| $L = SC + ST + SF$ | 86 | $\underline{-9{\cdot}0}$ |
| $L = SC + ST + SF + \underline{B}$ | 86 | $\underline{-9{\cdot}0}$ |
| $L = SC + ST + SF + \underline{B} + \underline{FC}$ | 87 | $\underline{-9{\cdot}0}$ |

Note: (a) Underline means not significant ($P > 0{\cdot}05$)
(b) Coefficients have been omitted for simplicity
(c) Key: C Cigarettes; L Lung cancer mortality (all ages); T Other tobacco; $L_0$ Lung cancer mortality (20–24 years); F Solid Fuel; S Stomach cancer mortality; B Bronchitis mortality

The regression equation for the multiplicative model was as follows:

$$L = \frac{C^{0{\cdot}76} \times F^{0{\cdot}3} \times S^{1{\cdot}2}}{10^{1{\cdot}9}}$$

The index for stomach cancer mortality was significantly greater ($P < 0{\cdot}05$) than the index for cigarette consumption. This further supported the importance of the association of stomach cancer mortality with lung cancer.

Bronchitis mortality did not appear to be an important factor overall. Its association with lung cancer mortality of particular countries might, however, be significant.

*The interpretation of stomach cancer mortality in its association with lung cancer*

It was reasonable to regard consumption of cigarettes, other tobacco products and solid fuel as measures of exposure of lung tissue to noxious products. The interpretation of stomach cancer mortality in the regression equation was not obvious but there appeared to be two general interpretations: one was to regard stomach cancer mortality as an index of exposure of lung tissue, the second as an index of susceptibility of lung tissue.

(i) *Stomach cancer mortality as an index of exposure.*—Doll (1955) has pointed out that national variations in the length of cigarette smoked probably contribute to international differences in the lung cancer/cigarette ratio. From estimates of butt length (Wynder and Hoffmann, 1967), and also on general grounds, it was reasonable to regard $\frac{1}{2}$ to $\frac{4}{5}$ as the likely extreme range of the mean length of cigarette smoked in different countries. This would give an extreme variation of about $1\frac{1}{2}$-fold in the ratio lung cancer/cigarette, assuming that the yield of carcinogenic products was linear with the length of cigarette smoked. The observed variation in the lung cancer/cigarette ratio was six-fold and suggested that the contribution of variations in length of cigarette smoked was a minor one.

Estimates of butt length during the period relevant to this study were available for only seven countries (Wynder and Hoffmann, 1967; Todd, 1963*b*). When cigarette consumptions were adjusted for the amount smoked the corrected value of the ratio lung cancer/cigarette remained positively correlated ($P < 0{\cdot}02$) with stomach cancer mortality. For this and the foregoing computation the yield of carcinogenic products was assumed to be linear with the length of cigarette smoked. Present evidence on the order of this dependence is conflicting (Wynder and Hoffmann, 1967). It it were shown subsequently that the yield of carcinogenic products increased non-linearly with butt length this would have to be taken into account. Butt lengths were found to be positively correlated ($P < 0{\cdot}02$) with national wealth estimated as the per capita Gross National Product (United Nations, 1950–65) for the year when butt lengths were estimated (17 estimates). As would be expected stomach cancer mortality was negatively correlated ($P < 0{\cdot}001$) with these estimates of wealth. Thus a consequence of a non-linear dependence might be that stomach cancer mortality represented, in its association with the lung cancer/cigarette ratio, mean butt length and so could be a measure of exposure of lung tissue.

(ii) *Stomach cancer as an index of susceptibility.*—Stomach cancer, in its association with lung cancer, could represent the susceptibility of lung tissue. Such an interpretation would give an equation of the form:

$$L = S(C + T + F).$$

For this equation $R^2$ was found to be 86% (Table IC). When stomach cancer mortality was included in the equation as a product with cigarette consumption only, appropriate if stomach cancer mortality represented butt length, $R^2$ was found to be 72% (Table IC). This lower value of $R^2$ supported the view that stomach cancer mortality represented susceptibility.

*Functional model for lung cancer induction*

The foregoing implicates five factors in the induction of lung cancer. A large number of different models are, of course, possible based on different combinations of these factors. Regression equations for several models were examined in an attempt to identify those which were reasonable on functional grounds and also gave a large multiple correlation coefficient, R.

Linear combinations (Table IB) gave a high value for R, but the constant term also was high. For this reason, and also on general functional grounds, this combination was regarded as unlikely. The constant term (a) was small in equations containing the product term SC (see Table IC). The inclusion of product terms SF and ST gave a higher value of R, while the constant term remained insignificant (Table IC). Evidence in support of this model has been described above, and the following was tentatively adopted as a likely model:

$$L = S(C + T + F).$$

Addition of a term for bronchitis mortality had essentially no effect on the value of the multiple correlation coefficient (Table IB and C). The partial regression coefficient for bronchitis was not significant (Table IC). When a product term FC was incorporated into the equation the partial regression coefficient was not significant and R was essentially unaltered (Table IC).

*Contribution of factors to lung cancer*

The regression equation for the adopted model was found to be:

$$L = \frac{S}{10^3}(22{\cdot}6\ C + 5{\cdot}46\ T + 0{\cdot}214\ F) - 9{\cdot}34$$

where L and S were annual mortality rates per $10^5$ males, C and T lb per adult per 15 years and F was $10^3$ kg. p.a. *per capita*.

Using the above equation the contributions of cigarettes, other tobacco products and solid fuel to lung cancer were estimated for each country. These are given in Fig. 2 as percentages of the total explained by the three factors. Also given are

FIG. 2.—Estimated contributions to lung cancer in males
For residuals, a positive value means that the predicted lung cancer rate exceeded the observed rate.

the residuals expressed as percentages of the observed lung cancer mortality rates. The average contributions for 20 countries were 70% from cigarettes, 10% from other tobacco and 20% from fuel consumption. The highest estimated contribution from fuel consumption was for West Germany (50%). The residuals were on average 15% of the observed lung cancer mortality rate. The three largest residuals were for Ireland, Canada and Norway.

*Lung cancer rates for females*

Data on consumption of cigarettes by females were not generally available and study of lung cancer rates in females was confined here to rates at 20–24 years. These rates were calculated for the period 1954–63 and on average were 43% of the corresponding rate for males. It was reasonable to assume that males and females had similar exposures to air pollution. The lower lung cancer rate in females aged 20–24 years could be accounted for, in terms of regression equations given in Table IA, by the lower mortality rates for stomach cancer and bronchitis in females compared with males.

### DISCUSSION

This study has indicated that three sources of respiratory carcinogens are important in the induction of lung cancer and as would be expected they are cigarettes, other tobacco and solid fuel. Evidence implicating cigarettes is extensive and widely accepted and studies have indicated that smoking of tobacco, other than cigarettes, appears to contribute relatively little to lung cancer (Doll and Hill, 1956). The partial regression coefficients of the model adopted in the present study were consistent with this finding. They indicated that the contribution of " other tobacco products " to lung cancer was one quarter of that for an equal weight of manufactured cigarettes. Consumption of " other tobacco products " included hand-made cigarettes and so, excluding tobacco consumed as cigarettes, the contribution of other tobacco would be less than one quarter.

Several studies have implicated air pollution in production of lung cancer (Daly, 1959; Stocks, 1967) and the urban-rural gradient of lung cancer mortality is generally attributed to it (Doll, 1955). Air pollution, estimated by solid fuel consumption, was found in the present study to be positively correlated ($P < 0{\cdot}001$) with lung cancer mortality in young males, aged 20–24 years. Lung cancer rates in this age group are not correlated with cigarette consumption (Hems, 1968*a*) and so air pollution might be a major cause of lung cancer in non-smokers. Considering lung cancer in the whole population the contribution of solid fuel consumption was, on average, 20%, compared with 70% attributable to cigarettes.

The three factors discussed above accounted for almost half of the variation in lung cancer mortality rates for males of all ages in 20 countries. When stomach cancer mortality was incorporated into the regression equation the amount of variation explained was increased to over 80%. This influence of the stomach cancer mortality rate would account for the apparently anomalous lung cancer rates of Austria, Finland, and the Netherlands, mentioned by Stocks (1967).

The association of lung cancer with stomach cancer could be accounted for in two general ways: stomach cancer might be associated with carcinogenic factors to which the lungs were exposed, or it might be associated with susceptibility of lung tissue. Because stomach cancer mortality and the mean length of cigarette smoked both appeared to be associated with poverty, stomach cancer mortality might indirectly be a measure of the mean length of cigarette smoked. However, if the yield of carcinogenic product was linear with the length of cigarette smoked it appeared unlikely that variation in butt length would account for more than one-quarter of the observed variation in the lung cancer/cigarette ratio. Furthermore, the best regression equation was one for which stomach cancer mortality would be interpreted as a measure of susceptibility of lung tissue. It is of interest to speculate that if stomach cancer mortality were regarded as an index of susceptibility to lung cancer, the diet, believed to be a major contributor to stomach cancer might influence lung tissue also. Any such effect of diet would be indirect, presumably mediated physiologically. Wynder and Hoffmann, (1967) pointed out that nutritional deficiencies might influence susceptibility to lung cancer. They cited work of Kreshover (1952, 1955) and Kreshover and Salley (1957, 1958) who found that a deficiency of the Vitamin B complex appeared to enhance susceptibility of rats to tobacco smoke condensates.

Lung cancer mortality rates in males aged 20–24 years appeared to be associated with bronchitis mortality, in addition to being associated with solid fuel consumption and stomach cancer mortality. It was tacitly assumed that conditions which influenced bronchitis mortality, the majority of which occurs in late adult life, were conditions which affected lungs of young adults also. Consideration of the nature of these conditions is beyond the scope of this study.

It was suggested in an earlier paper (Hems, 1968*a*) that the ratio lung cancer/cigarette was related to lung cancer in males aged 20–24 years. This is now seen as a consequence of their mutual association with stomach cancer mortality. It was suggested also (Hems, 1968*a*) that for females variations in the age dependence of lung cancer were complicated by the changing smoking habits of women. The age dependence might be further complicated by the change in susceptibility of females to stomach cancer which apparently occurs at middle age (Hems, 1968*b*) and which might, in the light of the present findings, be associated with a change in the susceptibility of lug tissue.

### CONCLUSIONS

1. For males of 20 countries consumption of cigarettes, other tobacco, and solid fuel explained almost half of the variation in lung cancer mortality. When stomach cancer mortality was included as an additional factor the part of the variation explained was increased to over 80%.

2. From inspection of regression equations a model was adopted which included interaction of stomach cancer mortality with cigarettes, other tobacco and solid fuel. With this model it was predicted that contributions to lung cancer were, on average for the 20 countries studied, 70% from cigarettes, 10% from other tobacco products and 20% from consumption of solid fuel.

3. Two interpretations of stomach cancer mortality, in its association with lung cancer, seem possible.

(i) Stomach cancer could, by virtue of its association with poverty, be an index of the length of cigarette smoked. If the yield of carcinogenic products was linear with the length of cigarette smoked, national differences in butt length would be unlikely to account for more than one quarter of the observed differences in the ratio lung cancer/cigarette. If, however, the yield of carcinogenic products increased non-linearly as butt length decreased then a greater part than one quarter could be explained. (ii) Stomach cancer mortality could be an index of susceptibility of lung tissue.

4. If stomach cancer mortality were interpreted as an index of susceptibility of lung tissue it is possible that diet, a cause of stomach cancer, might also influence the condition of lung tissue. Presumably this influence would be indirect and physiological.

5. Lung cancer in males aged 20–24 years was associated with solid fuel consumption and rates of mortality from stomach cancer and bronchitis. This association might apply to all non-smokers. Similar associations were found for lung cancer in females aged 20–24 years. Since rates of stomach cancer mortality and bronchitis mortality are lower in females these associations would account for the lower rate of lung cancer mortality amongst females in this age group.

The author wishes to acknowledge discussion of the paper with Professor E. Maurice Backett and also his indebtedness to Miss Alice Duncan for careful completion of the calculations on which this paper is based.

### REFERENCES

Daly, C.—(1959) *Br. J. prev. soc. Med.*, **13**, 14.

Doll, R.—(1955) *Adv. Cancer Res.*, **3**, 1.

Doll, R. and Hill, A. B.—(1956) *Br. med. J.*, ii, 1,071.

Hems, G.—(1968*a*) *Br. J. Cancer*, **22**, 466.—(1968*b*) *Br. J. Cancer*, **22**, 461.

Kreshover, S. J.—(1952) *J. Am. dent. Ass.*, **45**, 528.—(1955) *J. dent. Res.*, **34**, 798.

Kreshover, S. J. and Salley, J. J.—(1957) *J. Am. dent. Ass.*, **54**, 509.—(1958) *J. dent. Med.*, **3**, 130.

Segi, M. and Kurihara, M.—(1966*a*) ‘Cancer Mortality for Selected Sites in 24 Countries’ No. 4, Japan (Sendai).—(1966*b*) ‘Mortality for Selected Causes in 30 Countries’ (1950–1961), Japan (Sendai).

Stocks, P.—(1967) *Br. J. prev. soc. Med.*, **20**, 181.

Todd, G. F.—(1963*a*) *Tob. Res. Coun. Res. Pap.*, No. 6.—(1963*b*) *Tob. Res. Coun. Res. Pap.*, No. 5.

United Nations StatIsticaL Yearbook—(1950–65) United Nations.

Wynder, E. L. and Hoffman, D.—(1967) ‘Tobacco and Tobacco Smoke’. New York (Academic Press).